# Supplementary material for: Tracking tuberculosis control using detailed population health and satellite luminosity data: findings from Kazakhstan
Source: PLoS One. 2026 Apr 22;21(4):e0347191. doi: 10.1371/journal.pone.0347191 (PMC13102244; doi:10.1371/journal.pone.0347191)
Supplement: S1 Appendix — (DOCX) [file pone.0347191.s001.docx]

**S1 Appendix. Interpolation of missing years from QazStat.**

As noted, Kazakhstan’s Bureau of National Statistics [1] provides district-level age-sex distributions and ethnic compositions only after 2005, while 2000-2004 data are absent. We linearly interpolate the missing years using 1999 Census data [2].

However, these data are imperfectly aligned. Sex-specific data for 2005-2018 come in three bins. For males, these bins are years 0-15, 16-62, and 63+, while for females the bins are years 0-15, 16-57, and 58+. These classifications reflect children, working-age population, and retirees (women are eligible to retire five years earlier than men). In contrast the 1999 Census contains 16 bins for each sex: less than 1, 1-4, 5-9, 10-14, 15-19, 20-24, 25-29, 30-34, 35-39, 40-44, 45-49, 50-54, 55-59, 60-64, 65-69, and 70+. To align the Census and 2005-2018 QazStat data, we assume that populations are uniformly distributed within the 5-year bins.

**References**

1. Bureau of National Statistics. Chislennost' naseleniya Respubliki Kazakhstan po polu i otdel'nym vozrastnym gruppam [Population of the Republic of Kazakhstan by gender and age groups] [Internet]. Astana: Bureau of National Statistics; [n.d.] [cited 2025 Aug 20]. Available from: <https://stat.gov.kz/ru/industries/social-statistics/demography/>
2. Bureau of National Statistics of Republic of Kazakhstan (Qazstat). Naselenie Respubliki Kazakhstan po polu i vozrastu [Population of the Republic of Kazakhstan by gender and age] [Internet]. Astana: Bureau of National Statistics; 1999 [cited 2025 Aug 20]. Available from: <https://stat.gov.kz/ru/national/1999/>
