# Supplementary material for: Tracking tuberculosis control using detailed population health and satellite luminosity data: findings from Kazakhstan
Source: PLoS One. 2026 Apr 22;21(4):e0347191. doi: 10.1371/journal.pone.0347191 (PMC13102244; doi:10.1371/journal.pone.0347191)
Supplement: S2 Appendix — (DOCX) [file pone.0347191.s002.docx]

**S2 Appendix. Intercalibration Procedure**

Our intercalibration procedure follows Elvidge et al. [1] and requires selection of a reference satellite-year combination as well as a reference region. Following standard selection criteria (highest resolution photos and related year, along with regions that are stable and offer a wide range of observed values), we select the F18 satellite, the year 2010 and Bukhar-Zhyrauskiy and Pavlodarskiy districts as the reference region. Details appear in section 5.1 of Becker et al. [2].

Using the reference satellite-year and reference region, we fit a second order polynomial to adjust the Digital Numbers (DNs) in the reference region for the remaining satellite-year combinations. As a result of this procedure, we obtain a set of intercalibration coefficients for each satellite-year combination. Using these coefficients and uncalibrated DNs, we estimate the adjusted DNs for the remaining data according to:

$${DN}_{adj}= C_{0}+C_{1}*DN+C_{2}*{DN}^{2}$$

The intercalibration coefficients and comparison between the calibrated and uncalibrated data are discussed in section 5.1 of Becker et al. (2022).

**References**

1. Elvidge CD, Ziskin D, Baugh KE, Tuttle BT, Ghosh T, Pack DW, et al. A fifteen year record of global natural gas flaring derived from satellite data. Energies. 2009;2(3):595-622. Available from: <https://doi.org/10.3390/en20300595>
2. Becker C, Hill J, Muratov S. Brighter than a Million Suns: Contemporary Health Consequences of Atomic Testing in the Semipalatinsk Nuclear Polygon [Internet]. Durham (NC): Duke University, Department of Economics; 2022 [cited 2025 Aug 20]. Available from: <https://ucentralasia.org/media/0thnm34h/uca-ippa-wp70semipalatinskeng-1.pdf>
