# Supplementary material for: Tracking tuberculosis control using detailed population health and satellite luminosity data: findings from Kazakhstan
Source: PLoS One. 2026 Apr 22;21(4):e0347191. doi: 10.1371/journal.pone.0347191 (PMC13102244; doi:10.1371/journal.pone.0347191)
Supplement: S3 Appendix — (DOCX) [file pone.0347191.s003.docx]

**S3 Appendix. Details on matching results.**

**Description of Variables**

The variables used for PSM and presented in the figures in this document have the following interpretations:

*pct_0_15_male*– percent of district level population who are male and between 0-15 years old

*pct_16_62_male*– percent of district level population who are males and between 16-63 years (working age males)

*pct_63_plus_male*– percent of district level population who are males and older than 63 years (retired males)

*pct_0_15_female*– percent of district level population who are females and between 0-15 years old

*pct_16_57_male* – percent of district level population who are females and between 16-57 years (working age females)

*pct_58_plus_female*– percent of district level population who are females and older than 58 years (retired females)

*pct_0_15_total*– percent of district level population who are between 0-15 years old

*pct_63_58_total*– percent of district level population who are older than 63/58 years (63 for males, 58 for females – retired population)

*pct_male* – percent of district level population who are males

*MEAN* – mean of the district level night light luminosity

*STD* – standard deviation of district level night light luminosity

*VARIETY =* the number of unique digital numbers (luminosity values) for each district.

*pct_kaz* – percent of the district population that is ethnically Kazakh

*pct_rus* – percent of the district population that is ethnically Russian

*nurses_p10000* – district level number of nurses per 10,000

*phys_p10000* – district level number of physicians per 10,000

**Matching Quality**

*Zone of Extreme and Maximal Radiation Risk*

In this treatment group, 4 out of 57 observations lacked common support after the PSM procedure. Overall, among the zones of radiation exposure, the extreme and maximal radiation zone (ZEMR) shows the lowest quality of matching with mean and median bias around 19% and 16%, respectively. Rubin’s B is 29% and Rubin’s R is below 0.5. Heterogeneity of the age-sex structure and luminosity patters are the likely reasons for the poor quality of the matches. The results of the matching process are summarized below in Figure 1a.

**Figure 1a**

**ZEMR matching results**


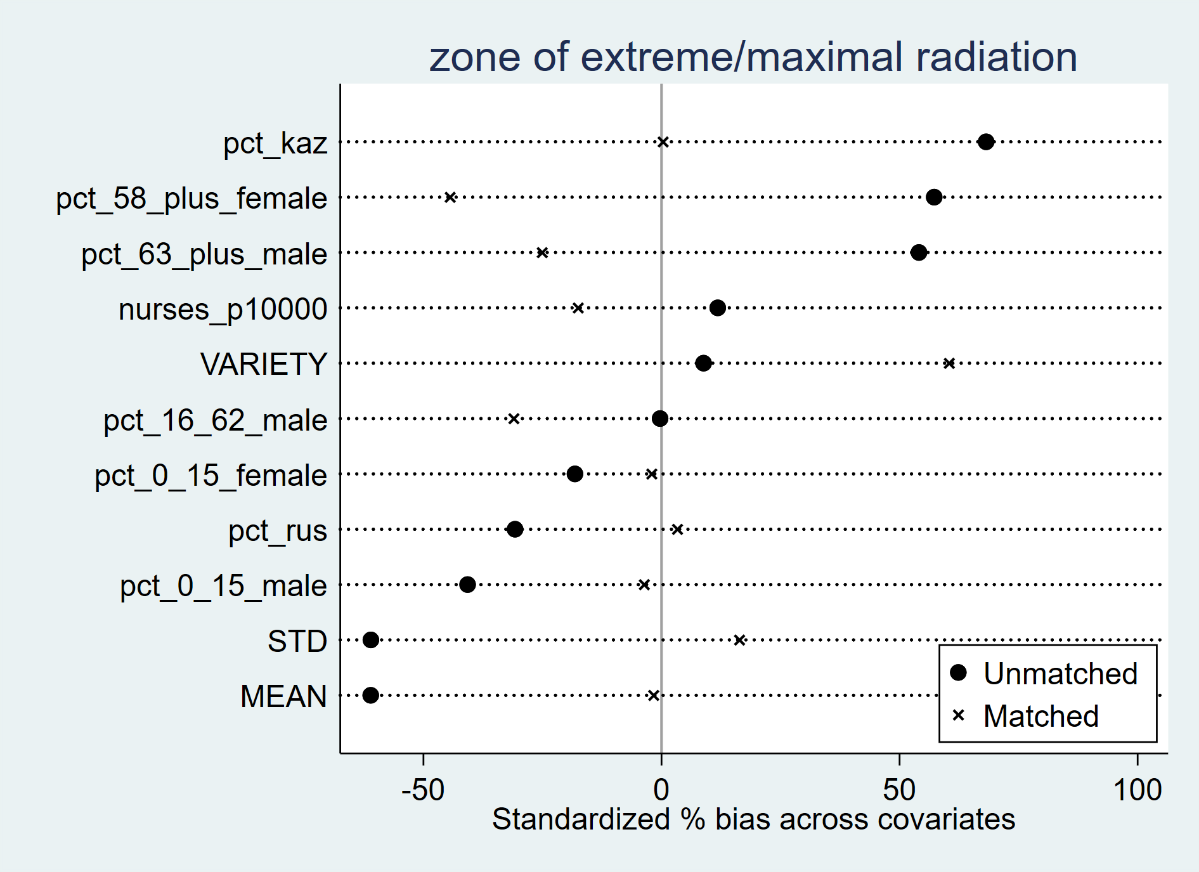


*Zone of High Radiation Risk*

Among the zones of radiation exposure, the zone of high radiation risk (ZHR) turned out to be the most heterogeneous. In this treatment group, 72 out of 190 observations were dropped due to the absence of common support between treatment and control groups. Interestingly, off-support observations are part of four districts: Oskemen and Ridder cities, and Glubokoe and Shemonaiha *raion*s. All of these localities have uncommon luminosity patterns and an atypical ethnic composition with a dominant Slavic population, which makes matching these districts to the rest of the Kazakhstan almost impossible. As for the remaining observations, Rubin’s B is at 48% and Rubin’s R is slightly below 0.5. Mean bias is at 11% while median bias is 9%. Matching results are summarized below in Figure 1b.

**Figure 1b**

**ZHR matching results**


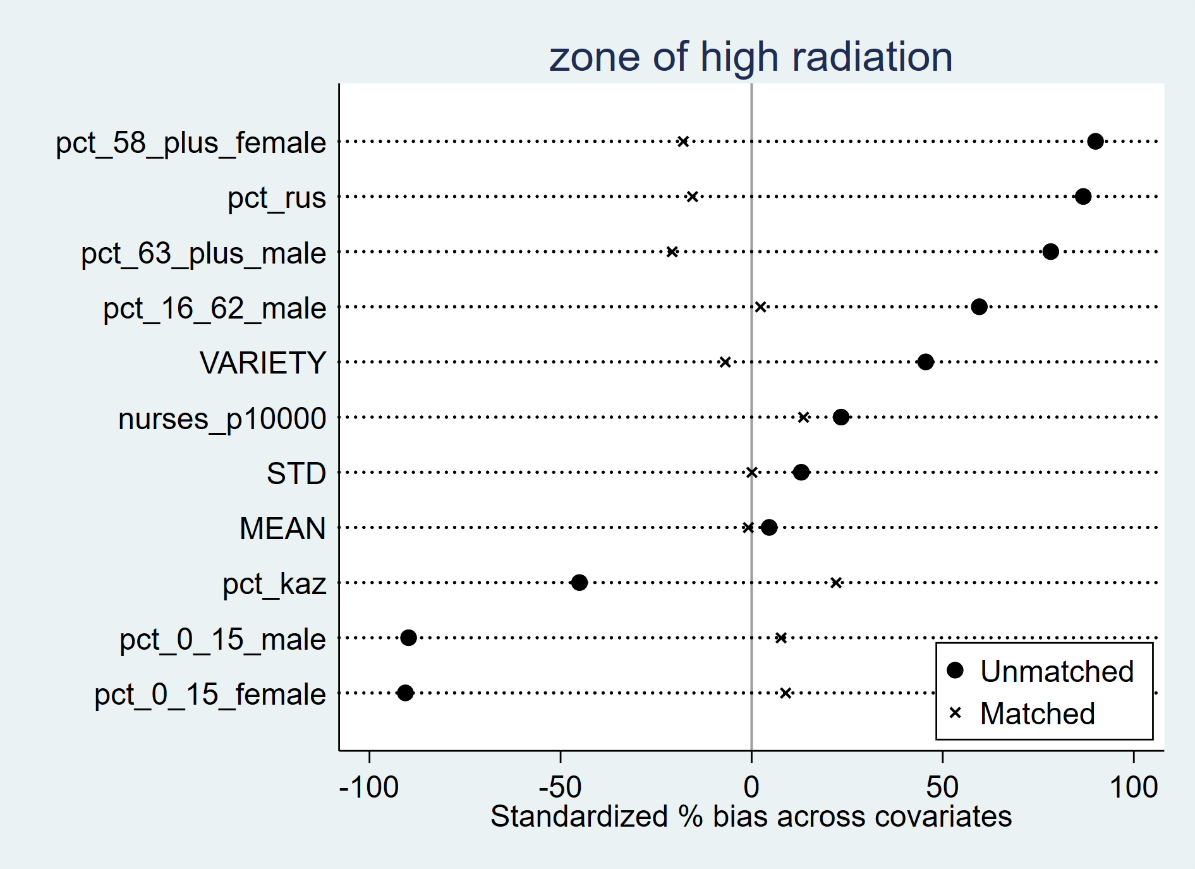


*Zone of Minimal Radiation Risk*

The best matching across the zones of radiation exposure is observed from the zone of minimal radiation risk (ZMR). Despite the fact that Rubin’s R is below 0.5, Rubin’s B is less than 25%. Moreover, the median and mean biases are close to 4%. After matching, only 2 out of 133 observations were dropped due to the lack of common support. The results of the PSM for zone of minimal radiation risk are shown below in Figure 1c. This zone does not contain big cities and is less ethnically diverse than the other two zones. These two factors can potentially explain why matching procedure was most successful in this zone.

**Figure 1c**

**ZMR matching results**


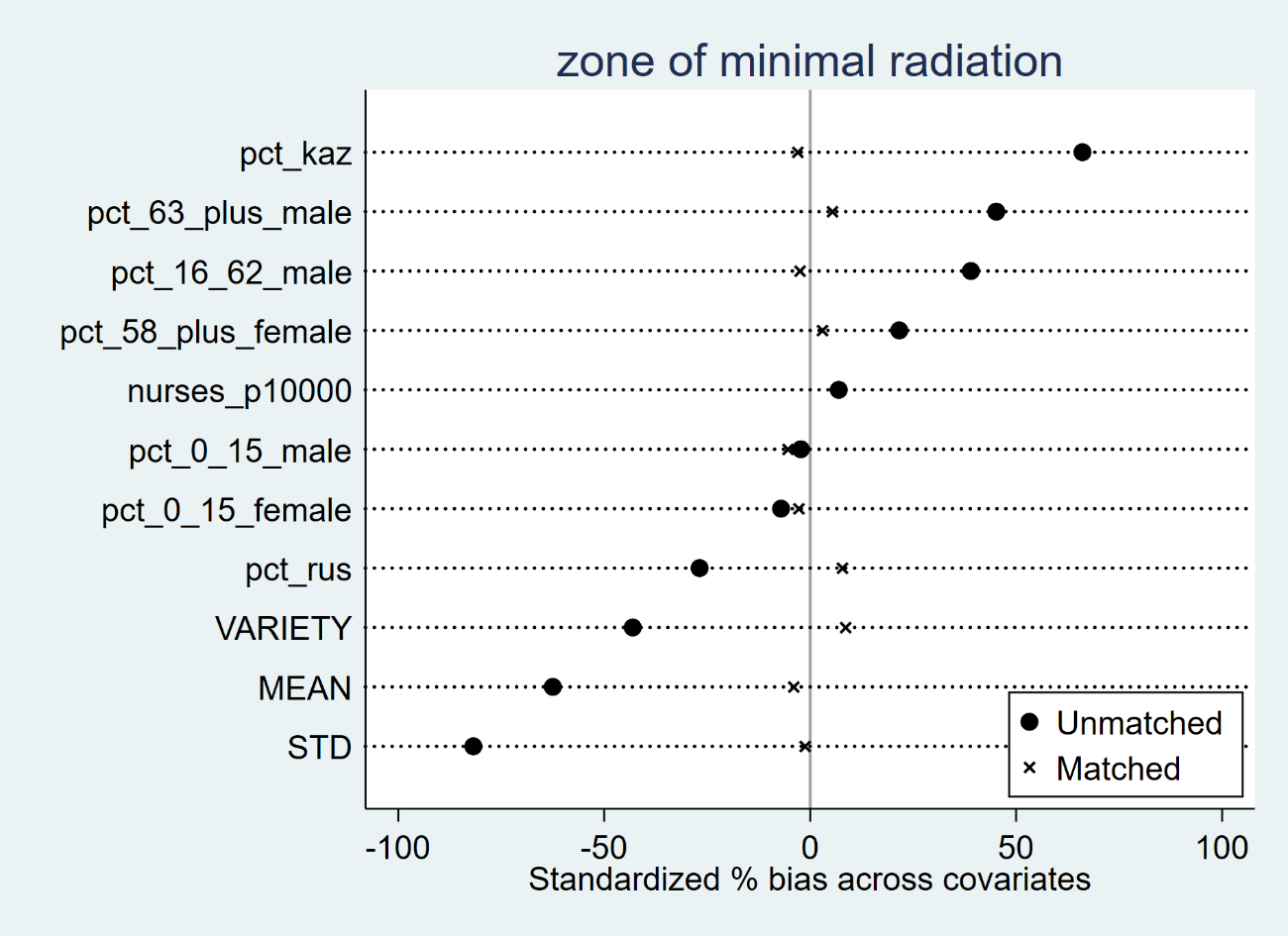


*Zone of Ecological Catastrophe*

Having discussed the zones of radiation risk, we now turn to the zones of ecological risk. Overall, matching quality for the zones of ecological risk is better than for the zones of radiation exposure. We believe this is primarily caused by using smaller set of control variables. One of the best matching results was achieved for the zone of ecological catastrophe (ECO_CAT). Although the Rubin’s R is below 0.5, Rubin’s B is only slightly greater than 25% (27%). The mean and median bias after matching are at 7% and 8%, respectively. Matching procedure was successful for all 57 observations in this group. Detailed results of the PSM procedure for the zone of ecological catastrophe are presented below in Figure 1d.

**Figure 1d**

**ECO_CAT matching results**

**
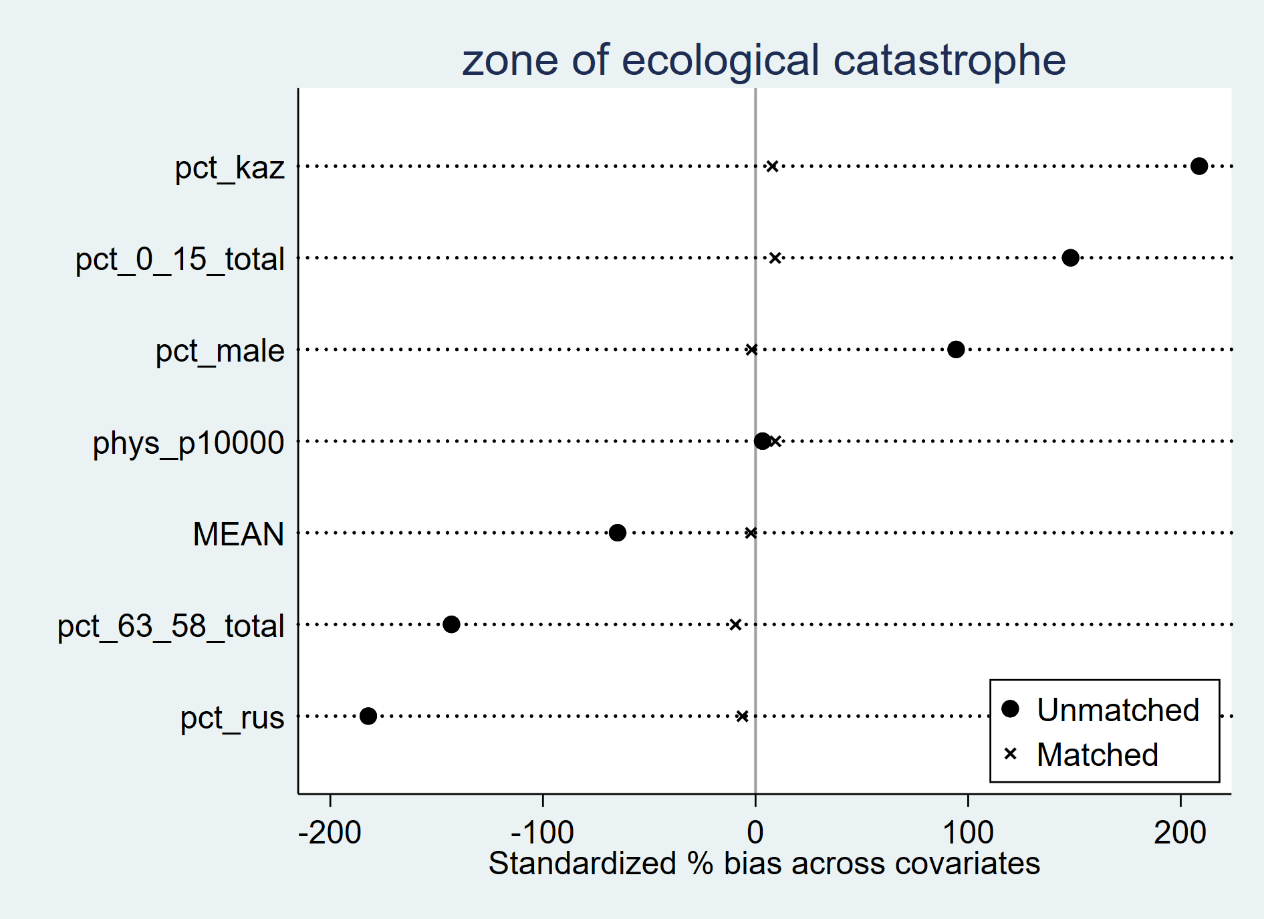
**

*Zone of Ecological Crisis*

Similar to the zone of ecological catastrophe, none of the 114 observations lacked common support after PSM procedure for the zone of ecological crisis (ECO_CRS). Despite the fact that Rubin’s B is 43% and Rubin’s R is below 0.5, mean and median bias were only 9% and 5%, respectively. Matching results are shown below in Figure 1e.

**Figure 1e**

**ECO_CRS matching results**

**
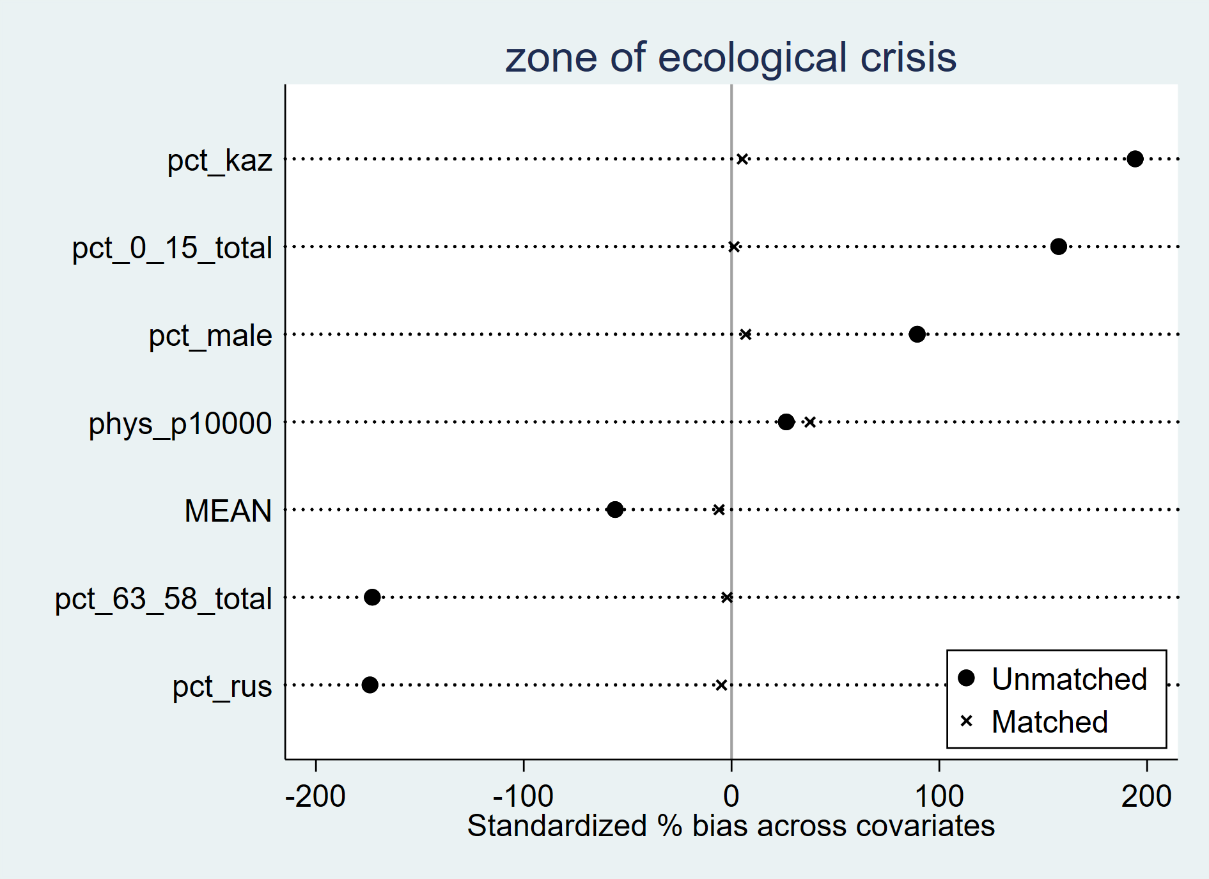
**

*Zone of Ecological Pre-Crisis*

Among the zones of ecological risk, the best matching results were obtained in the zone of ecological pre-crisis (ECO_PCR). Rubin’s R is around 0.25, while Rubin’s B is equal to 33%. Both median and mean bias are not greater than 5%. Out of 209 observations in this group, only 17 were lacking common support. All of these observations belong to the Turkistan city. This district has an abnormally low Russian population share, which could have complicated the matching procedure. Further details on the matching procedure are displayed in Figure 1f below.

**Figure 1f**

**ECO_PCR matching results**

**
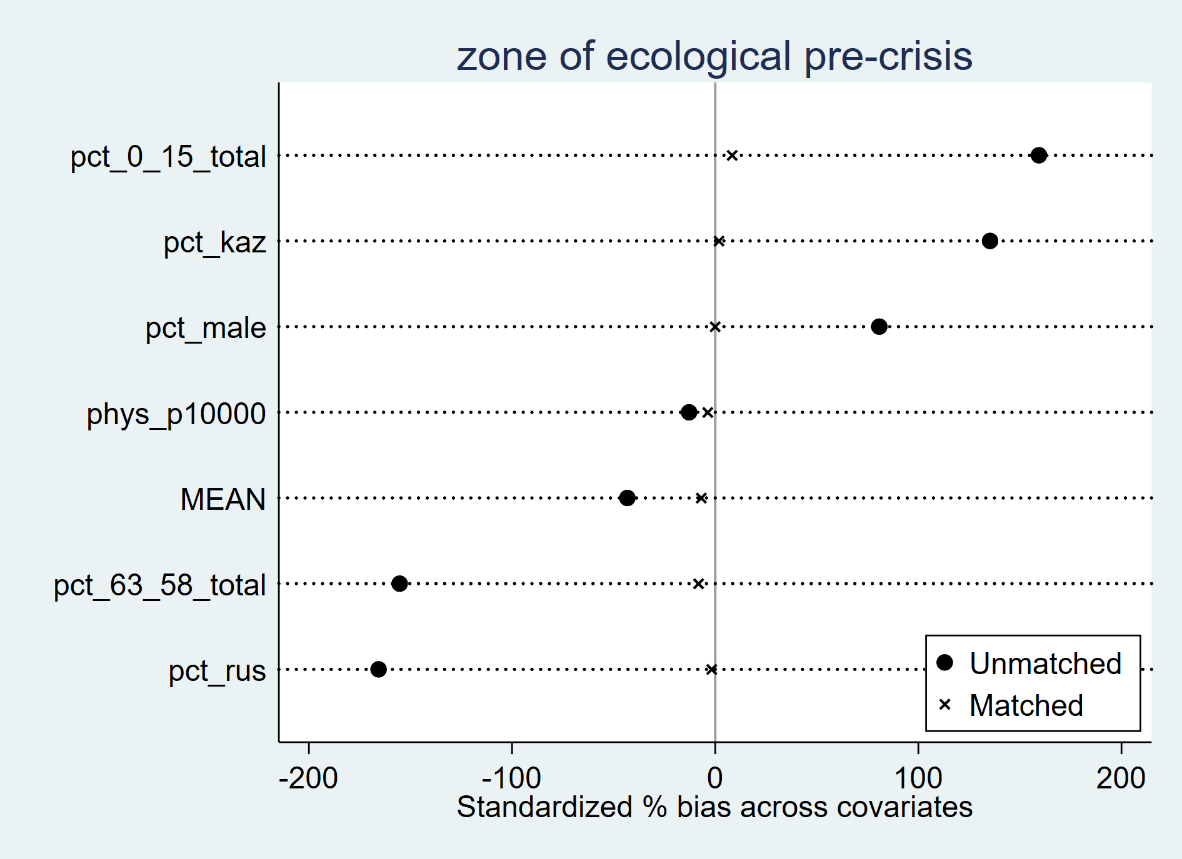
**

*Zone of Ecological Findings*

Lastly, we are left with the zone of ecological findings (ECO_FND). Here, Rubin’s B is around 15% while Rubin’s R is 1.44 in the matched sample. Median and mean bias are close to 7%. In this group, 10 out of 304 observations were absent of common support. Interestingly, all of the off-support observations belong to the Sairam *raion* of the Turkistan *Oblast*. The ethnic composition of this district is characterized by atypically low shares of both Kazakh and Russian populations and dominant share of Uzbek population, which may have resulted in a failure of the matching procedure. More detailed results are shown below in Figure 1f.

**Figure 1g**

**ECO_FND matching results**

**
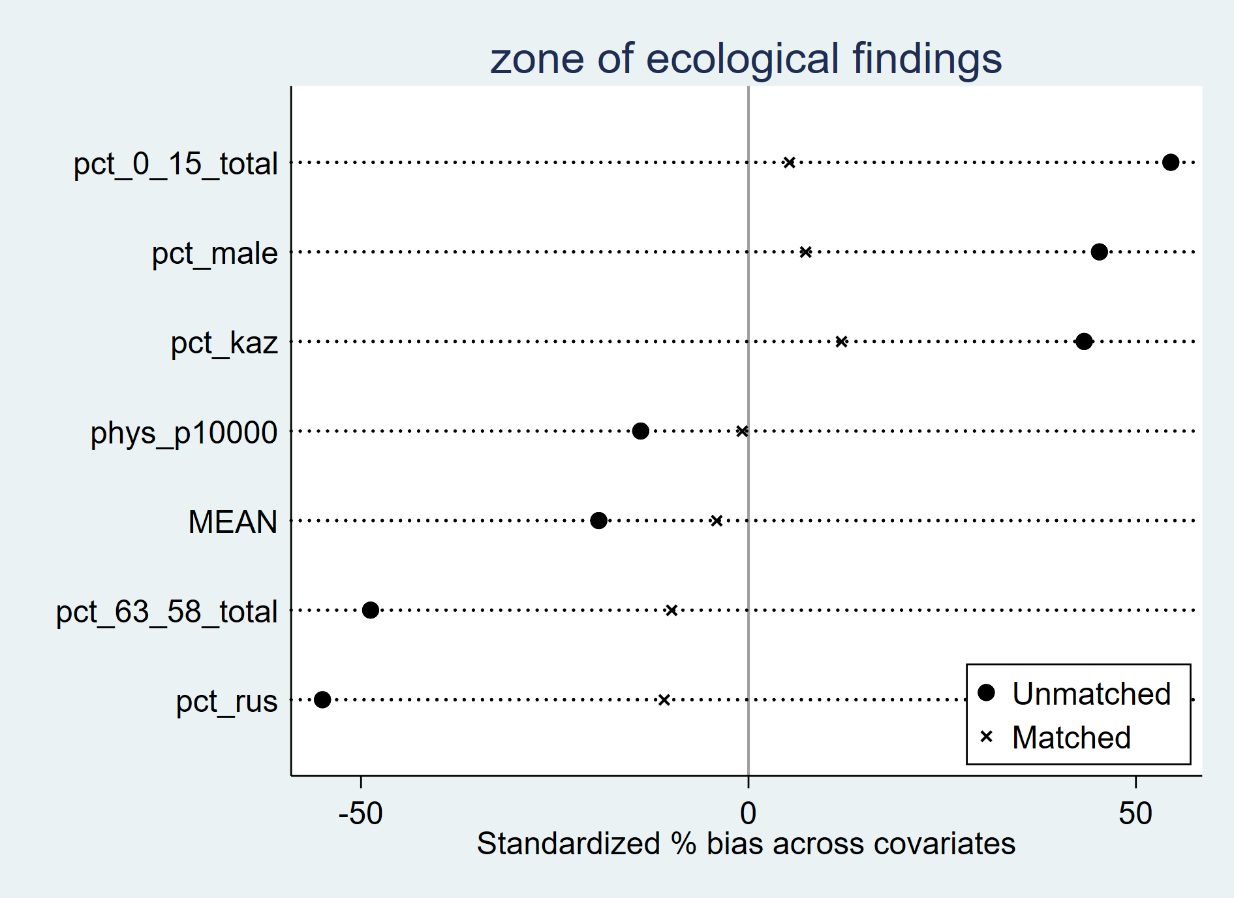
**
