# Supplementary material for: Tracking tuberculosis control using detailed population health and satellite luminosity data: findings from Kazakhstan
Source: PLoS One. 2026 Apr 22;21(4):e0347191. doi: 10.1371/journal.pone.0347191 (PMC13102244; doi:10.1371/journal.pone.0347191)
Supplement: S4 Appendix — (DOCX) [file pone.0347191.s004.docx]

**S4 Appendix. Detailed regression tables.**

Table of Contents

[Description of Models and Variables 2](#_Toc157296237)

[Section 1. General Regression Analysis 4](#_Toc157296238)

[Section 2. Time Trend Analysis 6](#_Toc157296239)

# Description of Models and Variables

This document contains regression coefficients and a description of the regressors. The part that contains regression coefficients is composed of two sections. Section 1 contains generalized regression results of a year fixed-effects model with clustered standard errors for TB prevalence and incidence over 2000-2018 period. Column 1 displays the findings for TB prevalence, while Column 2 represents the results for TB incidence.

Section 2 contains regression coefficients from the time trend analysis. Columns 1-3 display year fixed-effects model with clustered standard errors for TB prevalence on a restricted time domains – 2000-2005, 2006-2012 and 2013-2018, respectively. Columns 4-6 represent the same regression model over the same time intervals, but have TB incidence as a dependent variable.

The variables used as regressors have the following interpretations:

*pct_0_15_male*– percent of district level population who are male and between 0-15 years old

*pct_16_62_male*– percent of district level population who are males and between 16-63 years (working age males)

*pct_63_plus_male*– percent of district level population who are males and older than 63 years (retired males)

*pct_0_15_female*– percent of district level population who are females and between 0-15 years old

*pct_16_57_male* – percent of district level population who are females and between 16-57 years (working age females)

*pct_58_plus_female*– percent of district level population who are females and older than 58 years (retired females)

*STD* – standard deviation of district level night light luminosity

*MEAN* – mean of the district level night light luminosity

*MAX* – maximum of the district level night light luminosity

*SUM* – sum of the district level night light luminosity

*pct_rus* – percent of the district population that is ethnically Russian

*pct_ukr* – percent of the district population that is ethnically Ukrainian

# Section 1. General Regression Analysis

|  | (1) | (2) |
| --- | --- | --- |
| VARIABLES | TB prevalence | TB incidence |
|  |  |  |
| zemr | -16.94 | 2.554 |
|  | (20.53) | (10.06) |
| zhr | -20.41 | -3.825 |
|  | (24.24) | (7.162) |
| zmr | -25.96 | -8.127 |
|  | (30.22) | (8.439) |
| eco_cat | 101.6** | 30.48 |
|  | (46.86) | (19.94) |
| eco_crs | 128.2*** | 37.78*** |
|  | (28.14) | (10.46) |
| eco_pcr | 26.89 | 1.791 |
|  | (21.21) | (7.518) |
| eco_fnd | 9.327 | -8.672 |
|  | (20.47) | (6.363) |
| pct_0_15_male | -157.9 | -599.6 |
|  | (1,323) | (488.3) |
| pct_0_15_female | -711.3 | 483.9 |
|  | (1,390) | (510.1) |
| pct_16_62_male | -118.0 | 13.38 |
|  | (284.7) | (112.4) |
| pct_63_plus_male | -8,834*** | -2,893*** |
|  | (1,561) | (654.6) |
| pct_58_plus_female | 4,642*** | 1,356*** |
|  | (749.6) | (300.7) |
| STD | 0.271 | 0.330 |
|  | (2.158) | (0.761) |
| MEAN | -1.192* | -0.312 |
|  | (0.637) | (0.233) |
| MAX | 0.376 | 0.0805 |
|  | (0.502) | (0.207) |
| SUM | -0.000114 | -4.73e-07 |
|  | (0.000472) | (0.000187) |
| pct_rus | -270.6*** | -56.45*** |
|  | (56.69) | (20.62) |
| pct_ukr | 143.9 | -11.58 |
|  | (153.1) | (53.69) |
| _Iyear_2001 | 28.08*** | 10.62*** |
|  | (7.112) | (3.393) |
| _Iyear_2002 | 109.4*** | 23.99*** |
|  | (9.899) | (3.894) |
| _Iyear_2003 | 142.1*** | 23.49*** |
|  | (11.73) | (4.727) |
| _Iyear_2004 | 157.3*** | 17.12*** |
|  | (13.32) | (5.271) |
| _Iyear_2005 | 160.3*** | 9.694 |
|  | (15.62) | (6.204) |
| _Iyear_2006 | 144.9*** | -6.916 |
|  | (21.04) | (6.849) |
| _Iyear_2007 | -49.99*** | -23.70*** |
|  | (16.58) | (6.651) |
| _Iyear_2008 | -151.7*** | -32.25*** |
|  | (15.96) | (7.062) |
| _Iyear_2009 | -191.5*** | -60.81*** |
|  | (20.56) | (7.463) |
| _Iyear_2010 | -200.8*** | -67.19*** |
|  | (16.95) | (7.406) |
| _Iyear_2011 | -219.1*** | -78.19*** |
|  | (16.94) | (7.341) |
| _Iyear_2012 | -228.9*** | -82.94*** |
|  | (17.19) | (7.402) |
| _Iyear_2013 | -239.9*** | -91.45*** |
|  | (16.60) | (6.998) |
| _Iyear_2014 | -251.6*** | -97.69*** |
|  | (15.69) | (6.416) |
| _Iyear_2015 | -267.7*** | -104.3*** |
|  | (15.43) | (6.411) |
| _Iyear_2016 | -291.0*** | -110.5*** |
|  | (15.47) | (6.249) |
| _Iyear_2017 | -297.7*** | -109.5*** |
|  | (15.39) | (6.353) |
| _Iyear_2018 | -306.2*** | -114.1*** |
|  | (15.89) | (6.412) |
| Constant | 504.2*** | 175.8*** |
|  | (99.23) | (39.45) |
|  |  |  |
| Observations | 3,762 | 3,762 |
| R-squared | 0.630 | 0.542 |
| Year FE | Yes | Yes |
| Cluster SE | Yes | Yes |

Note: zemr, zhr and zmr, represent zones of extreme/maximal, high and minimal radiation exposure, respectively. eco_cat, eco_crs, eco_pcr and eco_fnd reflect zones of ecological catastrophe, crisis, pre-crisis, and findings, respectively.

***, **, and * denote significance at the 1%, 5%, and 10% levels,

respectively. Robust standard errors in parentheses.

# Section 2. Time Trend Analysis

|  | TB prevalence | | | TB incidence | | |
| --- | --- | --- | --- | --- | --- | --- |
| VARIABLES | (1)  Years 2000-2005 | (2)  Years  2006-2012 | (3)  Years  2013-2018 | (4)  Years  2000-2005 | (5)  Years  2006-2012 | (6)  Years  2013-2018 |
|  |  |  |  |  |  |  |
| zemr | -30.44 | 13.23 | -10.97 | 10.77 | 17.58 | -10.68 |
|  | (48.13) | (21.25) | (10.31) | (17.37) | (11.72) | (6.783) |
| zhr | -11.86 | -35.62* | -15.11 | -3.191 | 0.788 | -8.790* |
|  | (58.00) | (19.84) | (9.305) | (16.35) | (6.934) | (4.823) |
| zmr | -29.00 | -30.29 | -16.59 | -16.22 | 1.844 | -10.12** |
|  | (55.49) | (28.07) | (14.54) | (17.48) | (9.071) | (4.408) |
| eco_cat | 173.2* | 99.32*** | 40.71* | 84.95** | 11.14 | 2.275 |
|  | (91.87) | (35.88) | (24.09) | (33.16) | (19.23) | (9.199) |
| eco_crs | 186.0*** | 148.2*** | 50.16*** | 85.34*** | 20.12* | 8.235 |
|  | (40.00) | (38.01) | (13.64) | (19.01) | (11.33) | (5.845) |
| eco_pcr | 67.75 | 21.38 | 2.580 | 14.82 | -6.962 | -0.614 |
|  | (44.63) | (19.90) | (9.262) | (17.30) | (7.597) | (3.840) |
| eco_fnd | -13.97 | 36.00 | 3.635 | -15.30 | -7.926 | -1.391 |
|  | (34.86) | (28.33) | (8.819) | (11.81) | (6.601) | (4.153) |
| pct_0_15_male | 538.6 | 1,169 | -473.8 | -357.6 | -379.9 | -265.4 |
|  | (3,504) | (1,420) | (631.6) | (1,340) | (590.3) | (300.5) |
| pct_0_15_female | -2,394 | -1,266 | 570.8 | -83.84 | 358.8 | 538.1 |
|  | (3,563) | (1,618) | (701.8) | (1,381) | (686.1) | (339.1) |
| pct_16_62_male | -26.36 | 454.3 | 593.1* | 46.40 | 47.76 | 228.8* |
|  | (501.6) | (791.6) | (308.6) | (179.2) | (458.7) | (138.1) |
| pct_63_plus_male | -12,665*** | -8,825*** | -5,332*** | -4,379*** | -2,454*** | -1,133*** |
|  | (3,782) | (2,075) | (934.5) | (1,433) | (855.2) | (368.0) |
| pct_58_plus_female | 6,375*** | 5,418*** | 2,675*** | 1,851** | 1,156*** | 733.1*** |
|  | (1,923) | (1,011) | (523.4) | (734.9) | (412.4) | (225.6) |
| STD | 1.431 | 0.568 | -0.165 | 1.292 | 0.115 | -0.0392 |
|  | (4.297) | (2.225) | (0.774) | (1.535) | (0.888) | (0.378) |
| MEAN | -1.708 | -0.935 | -0.671** | -0.545 | -0.180 | -0.0742 |
|  | (1.297) | (0.696) | (0.274) | (0.475) | (0.265) | (0.119) |
| MAX | 0.352 | 0.282 | 0.564** | 0.186 | -0.0378 | 0.120 |
|  | (0.960) | (0.608) | (0.229) | (0.357) | (0.271) | (0.0956) |
| SUM | -0.000155 | -4.00e-05 | -0.000184 | -0.000226 | 0.000166 | 3.52e-05 |
|  | (0.000942) | (0.000582) | (0.000183) | (0.000344) | (0.000220) | (8.97e-05) |
| pct_rus | -459.1*** | -243.2*** | -81.83*** | -127.4*** | -22.63 | 0.856 |
|  | (127.9) | (54.65) | (29.52) | (44.32) | (21.59) | (13.31) |
| pct_ukr | 105.0 | 259.9 | 44.76 | 45.58 | -22.33 | -30.89 |
|  | (226.6) | (203.4) | (76.77) | (86.07) | (57.57) | (28.43) |
| 2001.year | 27.14*** |  |  | 9.922** |  |  |
|  | (8.879) |  |  | (3.894) |  |  |
| 2002.year | 107.8*** |  |  | 23.38*** |  |  |
|  | (14.37) |  |  | (5.347) |  |  |
| 2003.year | 139.8*** |  |  | 22.64*** |  |  |
|  | (19.46) |  |  | (7.014) |  |  |
| 2004.year | 154.3*** |  |  | 16.28* |  |  |
|  | (24.33) |  |  | (8.669) |  |  |
| 2005.year | 156.7*** |  |  | 8.874 |  |  |
|  | (30.06) |  |  | (10.75) |  |  |
| 2007.year |  | -195.2*** |  |  | -15.99*** |  |
|  |  | (15.43) |  |  | (2.762) |  |
| 2008.year |  | -298.2*** |  |  | -23.78*** |  |
|  |  | (13.53) |  |  | (3.806) |  |
| 2009.year |  | -339.2*** |  |  | -51.46*** |  |
|  |  | (17.45) |  |  | (4.813) |  |
| 2010.year |  | -347.8*** |  |  | -57.97*** |  |
|  |  | (15.04) |  |  | (4.844) |  |
| 2011.year |  | -367.1*** |  |  | -68.85*** |  |
|  |  | (15.58) |  |  | (5.277) |  |
| 2012.year |  | -378.0*** |  |  | -73.34*** |  |
|  |  | (16.55) |  |  | (5.599) |  |
| 2014.year |  |  | -9.546*** |  |  | -6.427*** |
|  |  |  | (3.053) |  |  | (1.404) |
| 2015.year |  |  | -24.86*** |  |  | -13.49*** |
|  |  |  | (3.208) |  |  | (1.702) |
| 2016.year |  |  | -46.81*** |  |  | -20.23*** |
|  |  |  | (4.292) |  |  | (2.084) |
| 2017.year |  |  | -52.05*** |  |  | -19.81*** |
|  |  |  | (4.290) |  |  | (2.434) |
| 2018.year |  |  | -59.53*** |  |  | -25.24*** |
|  |  |  | (5.253) |  |  | (2.935) |
| Constant | 660.1*** | 288.2 | -89.46 | 232.5*** | 139.7 | -57.89 |
|  | (104.0) | (392.0) | (155.0) | (37.06) | (224.4) | (71.82) |
|  |  |  |  |  |  |  |
| Observations | 1,188 | 1,386 | 1,188 | 1,188 | 1,386 | 1,188 |
| R-squared | 0.241 | 0.445 | 0.426 | 0.192 | 0.279 | 0.214 |
| Year FE | Yes | Yes | Yes | Yes | Yes | Yes |
| Cluster SE | Yes | Yes | Yes | Yes | Yes | Yes |

Note: zemr, zhr and zmr, represent zones of extreme/maximal, high and minimal radiation exposure, respectively. eco_cat, eco_crs, eco_pcr and eco_fnd reflect zones of ecological catastrophe, crisis, pre-crisis, and findings, respectively. ***, **, and * denote significance at the 1%, 5%, and 10% levels, respectively. Robust standard errors in parentheses.
