# Supplementary material for: Tracking tuberculosis control using detailed population health and satellite luminosity data: findings from Kazakhstan
Source: PLoS One. 2026 Apr 22;21(4):e0347191. doi: 10.1371/journal.pone.0347191 (PMC13102244; doi:10.1371/journal.pone.0347191)
Supplement: S5 Appendix — (DOCX) [file pone.0347191.s005.docx]

**S5 Appendix. Sensitivity Analysis results.**

**Sensitivity Analysis – PSM**

|  | After Propensity Score Matching | | | |
| --- | --- | --- | --- | --- |
| Zone of extreme/maximal radiation risk (ZEMR) | Treatment group  (n=54) | Matched Control group  (n=2698) | Difference | P-value |
| Prevalence of all forms of TB | 283.60 | 143.38 | 140.21 | 0.016 |
| Incidence of all forms of TB | 126.51 | 75.71 | 50.81 | 0.011 |
|  |  |  |  |  |
| Zone of high radiation risk (ZHR) | Treatment group  (n=117) | Matched Control group  (n=2698) | Difference | P-value |
| Prevalence of all forms of TB | 242.23 | 207.01 | 35.22 | 0.098 |
| Incidence of all forms of TB | 108.23 | 95.84 | 12.39 | 0.054 |
|  |  |  |  |  |
| Zone of minimal radiation risk (ZMR) | Treatment group  (n=132) | Matched Control group  (n=2698) | Difference | P-value |
| Prevalence of all forms of TB | 238.92 | 198.50 | 40.41 | 0.056 |
| Incidence of all forms of TB | 102.21 | 90.25 | 11.96 | 0.072 |

|  | After Propensity Score Matching | | | |
| --- | --- | --- | --- | --- |
| Zone of ecological catastrophe (eco_cat) | Treatment group  (n=57) | Matched Control group  (n=2698) | Difference | P-value |
| Prevalence of all forms of TB | 363.27 | 244.63 | 118.64 | 0.004 |
| Incidence of all forms of TB | 141.52 | 112.00 | 29.52 | 0.052 |
|  |  |  |  |  |
| Zone of ecological crisis (eco_crs) | Treatment group  (n=114) | Matched Control group  (n=2698) | Difference | P-value |
| Prevalence of all forms of TB | 364.48 | 280.70 | 83.78 | <0.001 |
| Incidence of all forms of TB | 144.80 | 119.59 | 25.21 | 0.005 |
|  |  |  |  |  |
| Zone of ecological pre-crisis (eco_pcr) | Treatment group  (n=192) | Matched Control group  (n=2698) | Difference | P-value |
| Prevalence of all forms of TB | 267.31 | 266.89 | 0.42 | 0.98 |
| Incidence of all forms of TB | 109.30 | 116.54 | -7.24 | 0.17 |
|  |  |  |  |  |
| Zone of ecological findings (eco_fnd) | Treatment group  (n=298) | Matched Control group  (n=2698) | Difference | P-value |
| Prevalence of all forms of TB | 274.37 | 258.87 | 15.50 | 0.29 |
| Incidence of all forms of TB | 104.97 | 111.59 | -6.62 | 0.06 |

**Sensitivity Analysis – Regressions**

|  | Model 1 | Model 2 |
| --- | --- | --- |
| VARIABLES | TB prevalence | TB incidence |
|  |  |  |
| zemr | -16.46 | 2.410 |
|  | (22.91) | (9.710) |
| zhr | -24.03 | -5.857 |
|  | (24.81) | (7.552) |
| zmr | -29.76 | -10.16 |
|  | (31.95) | (9.520) |
| eco_cat | 80.86* | 19.81 |
|  | (45.28) | (19.14) |
| eco_crs | 91.45*** | 18.93** |
|  | (23.06) | (8.349) |
| eco_pcr | 22.87 | -0.269 |
|  | (19.66) | (6.718) |
| eco_fnd | 7.815 | -9.383 |
|  | (20.50) | (5.877) |
| phys_p10000 | -0.165 | -0.0500 |
|  | (0.158) | (0.0587) |
| nurses_p10000 | 0.903*** | 0.456*** |
|  | (0.239) | (0.0866) |
| pct_0_15_male | -268.1 | -651.1 |
|  | (1,313) | (482.7) |
| pct_0_15_female | -634.3 | 521.5 |
|  | (1,384) | (505.7) |
| pct_16_62_male | 22.60 | 87.68 |
|  | (268.2) | (99.33) |
| pct_63_plus_male | -7,462*** | -2,177*** |
|  | (1,490) | (615.6) |
| pct_58_plus_female | 3,772*** | 904.5*** |
|  | (722.6) | (294.0) |
| STD | -0.849 | -0.279 |
|  | (2.015) | (0.691) |
| MEAN | -1.622** | -0.540** |
|  | (0.692) | (0.247) |
| MAX | 0.685 | 0.241 |
|  | (0.503) | (0.204) |
| SUM | -0.000118 | -1.21e-05 |
|  | (0.000455) | (0.000189) |
| pct_rus | -241.4*** | -40.73** |
|  | (55.68) | (20.17) |
| pct_ukr | 168.8 | 1.741 |
|  | (153.5) | (52.96) |
| _Iyear_2001 | 24.78*** | 8.948*** |
|  | (6.932) | (3.372) |
| _Iyear_2002 | 101.9*** | 20.15*** |
|  | (9.672) | (3.917) |
| _Iyear_2003 | 132.4*** | 18.53*** |
|  | (11.72) | (4.682) |
| _Iyear_2004 | 144.7*** | 10.66** |
|  | (13.22) | (5.258) |
| _Iyear_2005 | 144.4*** | 1.636 |
|  | (15.39) | (6.077) |
| _Iyear_2006 | 127.0*** | -16.06** |
|  | (21.65) | (6.699) |
| _Iyear_2007 | -67.06*** | -32.34*** |
|  | (16.24) | (6.524) |
| _Iyear_2008 | -169.5*** | -41.21*** |
|  | (15.89) | (6.918) |
| _Iyear_2009 | -209.7*** | -69.97*** |
|  | (20.75) | (7.227) |
| _Iyear_2010 | -220.7*** | -77.21*** |
|  | (16.85) | (7.171) |
| _Iyear_2011 | -239.7*** | -88.57*** |
|  | (17.00) | (7.120) |
| _Iyear_2012 | -250.3*** | -93.74*** |
|  | (17.15) | (7.163) |
| _Iyear_2013 | -257.6*** | -100.4*** |
|  | (16.38) | (6.808) |
| _Iyear_2014 | -267.8*** | -105.9*** |
|  | (15.55) | (6.273) |
| _Iyear_2015 | -284.6*** | -112.8*** |
|  | (15.50) | (6.337) |
| _Iyear_2016 | -306.9*** | -118.5*** |
|  | (15.58) | (6.181) |
| _Iyear_2017 | -317.4*** | -119.4*** |
|  | (15.74) | (6.435) |
| _Iyear_2018 | -323.2*** | -122.6*** |
|  | (16.03) | (6.443) |
| Constant | 433.0*** | 137.9*** |
|  | (92.32) | (34.44) |
|  |  |  |
| Observations | 3,760 | 3,760 |
| R-squared | 0.637 | 0.559 |
| Year FE | Yes | Yes |
| Cluster SE | Yes | Yes |
