# Supplementary material for: Tracking tuberculosis control using detailed population health and satellite luminosity data: findings from Kazakhstan
Source: PLoS One. 2026 Apr 22;21(4):e0347191. doi: 10.1371/journal.pone.0347191 (PMC13102244; doi:10.1371/journal.pone.0347191)
Supplement: Attachment — Submitted filename: Dear author.docx [file pone.0347191.s007.docx]

Dear author:

The paper is well written and presents a valuable contribution to the literature; however, a few amendments are required to improve clarity, methodological transparency, and overall presentation, as outlined below:

• The manuscript addresses an important and understudied topic by analyzing tuberculosis (TB) trends in Kazakhstan using both conventional health data and satellite nightlight (SNL) data as a proxy for economic development and data quality.

• The integration of satellite luminosity data with public health outcomes is innovative and contributes to methodological advancement in health geography and epidemiology.

• The use of regional and district-level data provides strong granularity, which is commendable. This level of detail enhances the robustness of the findings, particularly when comparing high-exposure areas to radiation and desiccation with national trends.

• The findings are relevant and timely, especially in the context of post-Soviet transitions and environmental degradation, which are often overlooked in global health literature.

• The abstract effectively summarizes the study; however, it could benefit from a clearer statement of the primary research question and hypothesis.

• While the methodology section outlines the use of matching and regression models, additional clarity is needed on the exact model specifications, control variables used, and rationale for the statistical techniques employed.

• It is not fully clear how completeness of TB data was assessed using SNL data. Further elaboration is recommended to validate this proxy's application.

• The discussion section makes appropriate connections to prior literature, but a more thorough critique of the study’s limitations, particularly related to measurement errors and potential confounding, would strengthen the manuscript.

• The ethics section states “N/A,” which may be appropriate given that no human subjects were directly involved. Nonetheless, a brief justification should be included in the manuscript to clarify why ethical approval was not necessary.

• The authors may wish to consider adding a visual representation or map overlaying TB data with SNL gradients to make spatial patterns more intuitive for readers.

• Although the paper is data rich, there is room for improving readability and accessibility for a broader audience, especially those less familiar with econometric techniques.

• Overall, this is a valuable contribution to the literature on environmental determinants of health and the utility of non-traditional data sources for public health surveillance.
