# Supplementary material for: Tracking tuberculosis control using detailed population health and satellite luminosity data: findings from Kazakhstan
Source: PLoS One. 2026 Apr 22;21(4):e0347191. doi: 10.1371/journal.pone.0347191 (PMC13102244; doi:10.1371/journal.pone.0347191)
Supplement: Attachment — Submitted filename: Response to Reviewers.docx [file pone.0347191.s008.docx]

**Dear Editor and Reviewers,**

We would like to thank you for valuable comments and insightful feedback! We carefully reviewed your responses and did our best to address every point that was raised during the revision.

**Response to Points raised by Academic Editor:**

1. To the best of our knowledge, we have updated the manuscript to fit into PLOS ONE’s style requirements, including those for file naming.
2. To our knowledge, all authors are included in the submitted files. There may have been a confusion between “Charles Becker” and “Charles Maxwell Becker”. This is the same person, where “Maxwell” is a middle name. The paper was reviewed to ensure the consistency between author names.
3. Please see the answer for the Point 2.
4. Figures 1 and 2 were generated on a basis of a MedInform dataset. The dataset itself is a part of a publicly available MedInform DPS program that contains district-level data and allows for a generation of spatial images (<http://www.medinfo.kz/#/dpsraion> ). We obtained images for the Figure 1 and 2 using this program and modified them a little in Microsoft Paint to include Figure title and the name of the Oblast (Kyzylorda). If necessary, we can provide a note in the paper, specifying the origin of the Figures 1 and 2.

As for the Figure 3, it was generated on a basis of a publicly available district-level map of Kazakhtan using ArcMap program (<https://data.humdata.org/dataset/kazakhstan-administrative-boundaries-taxonomy> ). Then, zones were colored based on a publicly available definition of “treated” areas, and later this figure was modified in Microsoft Paint to include the names of the treated areas.

Thus, we believe, there are no restrictions on publishing Figures 1-3.

5. Captions are supplied in the “Supporting Information” section in the end of the manuscript. Appendix files are renamed accordingly.
6. Our dataset contains aggregated information at district-level. There is no personal or individual-level information in the dataset.
7. The files “data file for TB paper PLOS One.dta” and “do file for TB paper PLOS One.do” are Stata files. All of the econometric analysis in the paper was carried out in the Stata program.

Since we carried out a “Sensitivity Analysis”, we have updated our data files accordingly. All of the data files are now provided in “S1 File. Supplementary Data Files”. This file contains our dataset in Stata (dta format), its copy in Excel, Stata do-file as well as its copy in TXT format.

8. References (both in paper and appendices) were updated according to PLOS One requirements.

**Response to Points raised by Reviewer 1:**

Since the commentaries are not numbered, our answers will be referring to the order of the points as they appear in the Reviewer’s responses.

Points 1-4 and Point 12 require no actions upon the authors.

Point 5: Abstract wording was modified and clarity improved.

Point 6: Additional clarification on econometric techniques, rationale and some of the variables is provided in the paper.

Point 7: It may have been unclear (I think that Reviewer 1 refers to the Abstract) about how completeness of TB data is related to SNL data. In fact, there is no direct relationship between completeness of TB data and SNL data. We use SNL data as a proxy of economic development/activity since there is no publicly available information on district GDP for the whole period of interest. We use SNL data as a control for economic development of the district, since literature indicates that SNL data can serve as a proxy for economic activity.

Point 8: Study limitations are now thoroughly discussed in a “Limitations” sub-section, which is placed in “Methods” section (right after “Propensity Score Matching” and “Regression Analysis”)

Point 9: A brief explanation of why no ethical approvals were required is provided in the paper (“Data” section, “MedInform” sub-section).

Point 10: As mentioned in the response to Point 7 we do not aim to establish any relationship between SNL data and completeness of TB data. For this reason, we believe that adding this type of maps is unnecessary.

Point 11: We believe that above-mentioned modifications of the paper improved its readability and accessibility for broader audience. We also did some minor improvements to the “Introduction” section to highlight some earlier studies and improve the transition to the scope of the paper.

**Response to Points raised by Reviewer 2:**

1. We have conducted a sensitivity analysis check with the PSM results by omitting healthcare quality variables from the list of controls. A short “Sensitivity Analysis” section was added into the paper where we discuss changes in PSM results. PSM tables are provided in the Appendix 5.
2. Similarly, to PSM, we also conducted a sensitivity analysis of Regression findings. Here, contrary to PSM, we added healthcare quality measures into regressions and re-estimated them. Updated regressions are briefly discussed in the “Sensitivity Analysis” section, while full regression tables are supplied in the Appendix 5.
3. No action is required
4. Stylistic and wording changes suggested by Reviewer 2 were implemented in the paper. References are formatted according to the PLOS One requirements.
