# Supplementary material for: Tracking tuberculosis control using detailed population health and satellite luminosity data: findings from Kazakhstan
Source: PLoS One. 2026 Apr 22;21(4):e0347191. doi: 10.1371/journal.pone.0347191 (PMC13102244; doi:10.1371/journal.pone.0347191)
Supplement: Attachment — Submitted filename: Response_to_Reviewers_auresp_2.docx [file pone.0347191.s009.docx]

**Dear Editor and Reviewers,**

We would like to thank you for valuable comments and insightful feedback! We carefully reviewed your responses and did our best to address every point that was raised during the revision. Also, we made minor corrections to several grammatical/verbal/punctuation errors found in the manuscript (highlighted in yellow as well)

**Response to Points raised by Reviewer 3:**

Point 1. Throughout the paper, we replaced administrative division names with their respective English terms, namely we changed “raion” for district, and “obalst” for province.

Point 2. We provided more detail about extended OLS series with sources that utilize the data. Changes were made in “Data – DMSP-OLS” section and References.

Point 3. We added a footnote (In Table 2) explaining our reasoning behind the use of several nighttime lights metrics and mentioned that, in principle, one metric could have been used.

Point 4. We expanded the “Limitations” section to account for the point raised by the reviewer.

Point 5. We utilize nighttime light metrics as a proxy for economic development to make more robust matching and regression results. Following your comment, we decided to reduce the focus on nighttime lights metrics, and modified our Abstract and Conclusion to be in line with the results of the paper.

**Response to Points raised by Reviewer 4:**

Point 1. We substantially reviewed and rewritten the abstract to focus more clearly on methodology and main findings.

Point 2. We provided more detail in the Results section (Zones of radiation risk – Regression Analysis) and explained why we observed differences between PSM and regression findings. We also made minor changes throughout the paper to provide more careful discussion of the continued relevance of radiation risk.
